# *TERT* promoter mutations are a rare event in gastrointestinal stromal tumors

**DOI:** 10.1186/s40064-015-1606-2

**Published:** 2015-12-30

**Authors:** Keisuke Akaike, Midori Toda-Ishii, Yoshiyuki Suehara, Kenta Mukaihara, Daisuke Kubota, Keiko Mitani, Tatsuya Takagi, Kazuo Kaneko, Takashi Yao, Tsuyoshi Saito

**Affiliations:** Department of Human Pathology, Juntendo University, School of Medicine, 2-1-1, Hongo Bunkyo-ku, Tokyo, 113-8421 Japan; Department of Orthopedic Surgery, Juntendo University, School of Medicine, 2-1-1, Hongo Bunkyo-ku, Tokyo, 113-8421 Japan

**Keywords:** Gastrointestinal stromal tumor, Telomere dysregulation, *TERT* promoter, ATRX, DAXX

## Abstract

Recently, the impact of telomere dysregulation on malignant progression has been reported in many cancers. A few studies have examined *TERT* promoter mutations in gastrointestinal stromal tumors (GISTs). Irregular telomerase activation can be maintained by *TERT* hot spot alterations and alternative lengthening of telomeres (ALT) characterized by inactivation of either the alpha-thalassemia/mental retardation syndrome X-linked (ATRX) or death domain–associated protein (DAXX). To elucidate the clinicopathological impact of telomere dysregulation in GISTs, we examined 92 cases of GISTs for *TERT* promoter hot spot mutations along with immunohistochemical analysis of ATRX and DAXX expression, and compared these findings with the clinicopathological features. Univariate clinicopathological analysis revealed that tumor site, smaller tumor size, presence of necrosis, higher mitotic rate (>5/50 high-power fields) and risk classification were prognostic factors for either disease-free survival or overall survival. Two of 92 informative cases (2.2 %) were found to have heterozygous *TERT* promoter mutations (C228T), and these mutations occurred in a low-risk and a high-risk tumor, respectively. On immunohistochemical analysis for ATRX and DAXX, 16 (17.4 %) and 3 (3.3 %) of 92 cases showed loss of expression of ATRX and DAXX, respectively. Loss of expression of ATRX and DAXX were mutually exclusive except for one case. *TERT* promoter mutations were also mutually exclusive of the ALT phenotype. Telomere dysregulation was not associated with patient survival; however, telomere dysregulation was frequently observed in tumors of extra-gastric origin, which have an adverse outcome compared to those of gastric origin.

**Electronic supplementary material:**

The online version of this article (doi:10.1186/s40064-015-1606-2) contains supplementary material, which is available to authorized users.

## Background

Gastrointestinal stromal tumors (GISTs) are the most common mesenchymal tumors of the digestive tract occurring in stomach (50–60 %), small intestine (30–35 %), colon and rectum (5 %), esophagus (<1 %), and other locations, respectively (Joensuu et al. 2012, 2013). GISTs have varying malignant potential ranging from benign small tumors to high-grade sarcomatous neoplasms (Joensuu et al. 2012, 2013).

Gastrointestinal stromal tumors are characterized by genetic alterations of the activating tyrosine-kinase receptor, *KIT* (found in 80 % of tumors), and *PDGFRA* (found in approximately 10 % of tumors) (Corless et al. 2004). *KIT* and *PDGFRA* are considered key oncogenes in GIST pathogenesis. Imatinib, a tyrosine kinase inhibitor of KIT and platelet-derived growth factor receptor alpha, is administered as standard adjuvant therapy to prevent recurrence and metastases in GISTs with high malignant potential (Dagher et al. 2002; Dematteo et al. 2009).

Because treatment decisions depend upon it, it is important to be able to accurately estimate the risk for recurrence of GISTs after surgical resection. Conventional risk classification systems are based on clinicopathological features (Joensuu et al. 2012; Miettinen and Lasota 2006; Fletcher et al. 2002). Genetic and proteomic analysis has revealed molecular biomarkers that are useful for predicting the malignant grade and prognosis of GISTs (Suehara et al. 2008; Kubota et al. 2013).

A previous study has reported the impact of telomere dysregulation on aggressive behavior in many malignancies (Shay and Bacchetti 1997). Telomeres consist of repetitive DNA sequences, predominantly with TTAGGG hexanucleotide DNA sequences (Shay and Bacchetti 1997). Cancer cells maintain the lengths of their telomeres through various mechanisms in order to prevent critical telomere shortening and can therefore sustain a limitless replicative potential. Two mechanisms of telomere maintenance have been identified: telomerase activation and alternative lengthening of telomeres (ALT) (Reddel 2014).

Telomerase activation is regulated by telomerase reverse transcriptase (TERT), a catalytic subunit of the telomerase complex. Recently, recurrent ‘hot spot’ mutations in the promoter region of *TERT* have been reported in melanomas (Horn et al. 2013), primary nervous system tumors (Koelsche et al. 2013), thyroid carcinomas (Landa et al. 2013), hepatocellular carcinomas (Nault et al. 2013), solitary fibrous tumors (Akaike et al. 2015), and bone and soft tissue sarcomas (Saito et al. 2016). These mutations result in the creation of a new binding site for E-twenty-six (ETS)/ternary complex factor (TCF) and increased *TERT* transcriptional activity (Horn et al. 2013).

Alternative lengthening of telomeres regulates the length of telomeres in 10–15 % of cancers, and ALT-positive tumors are characterized by marked telomerase-independent telomere length heterogeneity (Cesare and Reddel 2010). Several studies have revealed that the ALT-positive phenotype correlates perfectly with the inactivation of alpha-thalassemia/mental retardation syndrome X-linked (ATRX) or death domain–associated protein (DAXX) in pancreatic neuroendocrine tumors (PanNETs), astrocytomas, and leiomyosarcomas (Heaphy et al. 2011; Marinoni et al. 2014; Abedalthagafi et al. 2013; Liau et al. 2015). ATRX and DAXX form a chromatin-remodeling complex and are required for histone H3.3 deposition and remodeling of telomeres (Lewis et al. 2010).

A few researchers have identified *TERT* promoter mutations in GISTs (Campanella et al. 2015; Killela et al. 2013; Vinagre et al. 2013); however, ALT through ATRX and DAXX protein inactivation has not been reported in GISTs and the clinicopathological impact of telomere dysregulation in GISTs remains unknown.

In this study, we investigated telomere dysregulation as estimated by *TERT* promoter mutations and loss of expression of either ATRX or DAXX, and examined the correlation between these changes and clinicopathological features of patients with GISTs.

## Methods

### Sample preparation

The records of 92 patients with primary GISTs were retrospectively collected from the Department of Pathology, Juntendo University Hospital, Japan. All patients had been treated at the Juntendo University Hospital between 2000 and 2013. This study was approved by the research ethics committee of our institution. All cases were primary tumors and surgically resected specimens: 90 cases were obtained via total resection and two via partial resection (cases #24, #28) due to the large tumor size not being suitable for total resection.

A diagnosis of GISTs was made according to the World Health Organization (WHO) Classification of Tumors of Soft Tissue and Bone (Fletcher et al. 2013). To confirm a diagnosis of GISTs, we used immunohistochemical staining for c-KIT (CD117 antibody, DAKO Japan Corp., Tokyo, Japan) and DOG1 (mouse monoclonal, K9, Leica Biosystems) proteins. The tumor size, presence of necrosis, mitotic rate, and MIB-1 index were obtained for each case (Hamilton et al. 2000). Risk classification was based on the modified NIH consensus classification according to tumor size, tumor location and mitotic activity (Joensuu et al. 2012). Fifteen cases had liver metastasis, two of which were present at diagnosis. Three cases had local recurrence, two of which also had liver metastasis. The follow-up period ranged from 0.3 to 186 months (median 59.0 months; mean 67.1 months).

### Mutational analysis of the *TERT* promoter

Genomic DNA was extracted from each formalin-fixed paraffin-embedded (FFPE) block with the QIAamp DNA FFPE Tissue Kit (Qiagen, Germany). The polymorphism at the two-mutational hot spot of the *TERT* promoter region (1,295,228 C > T and 1,295,250 C > T; termed C228T and C250T, respectively) was examined using polymerase chain reaction (PCR), followed by direct sequencing. PCR was performed using AccuPrime™ GC-Rich DNA Polymerase (Invitrogen, Germany) according to the manufacturer’s protocol as follows: initial denaturation at 95 °C for 3 min and 40 cycles of 95 °C for 30 s, 55 °C for 30 s, 72 °C for 1 min, followed by 72 °C for 10 min. The primer sequences were forward: 5′-AGTGGATTCGCGGGCACAGA-3′ and reverse: 5′-CAGCGCTGCCTGAAACTC-3′. The PCR product was electrophoresed on a 2 % agarose gel. The PCR products with the appropriate sizes were excised from the gel and subsequently sequenced. For cases with a *TERT* promoter mutation, the corresponding normal tissue was also examined for the presence of the mutation to confirm that the detected mutation was somatic in nature.

### Immunohistochemical analysis for ATRX and DAXX

Immunohistochemical staining was performed for ATRX and DAXX using formalin-fixed, paraffin-embedded (FFPE) tissues according to the streptavidin–biotin method. Briefly, 4-μm thick tissue sections were autoclaved in 10 mM TE buffer (pH 6.0) at 121 °C for 30 min, and then incubated with anti-ATRX (Rabbit polyclonal, ATLAS Inc., Stockholm, Sweden, 1:500 dilution) and anti-DAXX (Rabbit polyclonal, SIGMA Life Science, St Louis, MO, USA, 1:500 dilution) antibodies at 4 °C overnight. The stained tissues were assessed by two of the authors (K.A. and T.S.) without prior knowledge of the clinical information. Nuclear staining was considered positive for ATRX and DAXX, and complete negative staining throughout the tumor tissue despite positive staining in vascular endothelial cells was interpreted as a loss of expression. Because loss of expression of either ATRX or DAXX has been shown to correlate well with the ALT phenotype in a previous study (Jiao et al. 2011), cases with loss of expression in either ATRX or DAXX were considered to exhibit telomere dysregulation similar to cases with *TERT* promoter mutated tumors.

### Statistical analysis

We defined any cases with *TERT* promoter hot spot mutations or the loss of nuclear expression of either ATRX or DAXX as positive for telomere dysregulation. The Mann–Whitney U test and the Chi-square test were used to examine associations between any clinicopathological features and telomere dysregulation. The impact of telomere dysregulation on disease-free survival (DFS) or overall survival (OS) rate was calculated by using Kaplan–Meier analysis with the log-rank test. Multivariate analyses were performed using a Cox proportional-hazards regression model of the survival rates.

## Results

### Clinicopathological features of 92 cases of GISTs

**Fig. 1:**
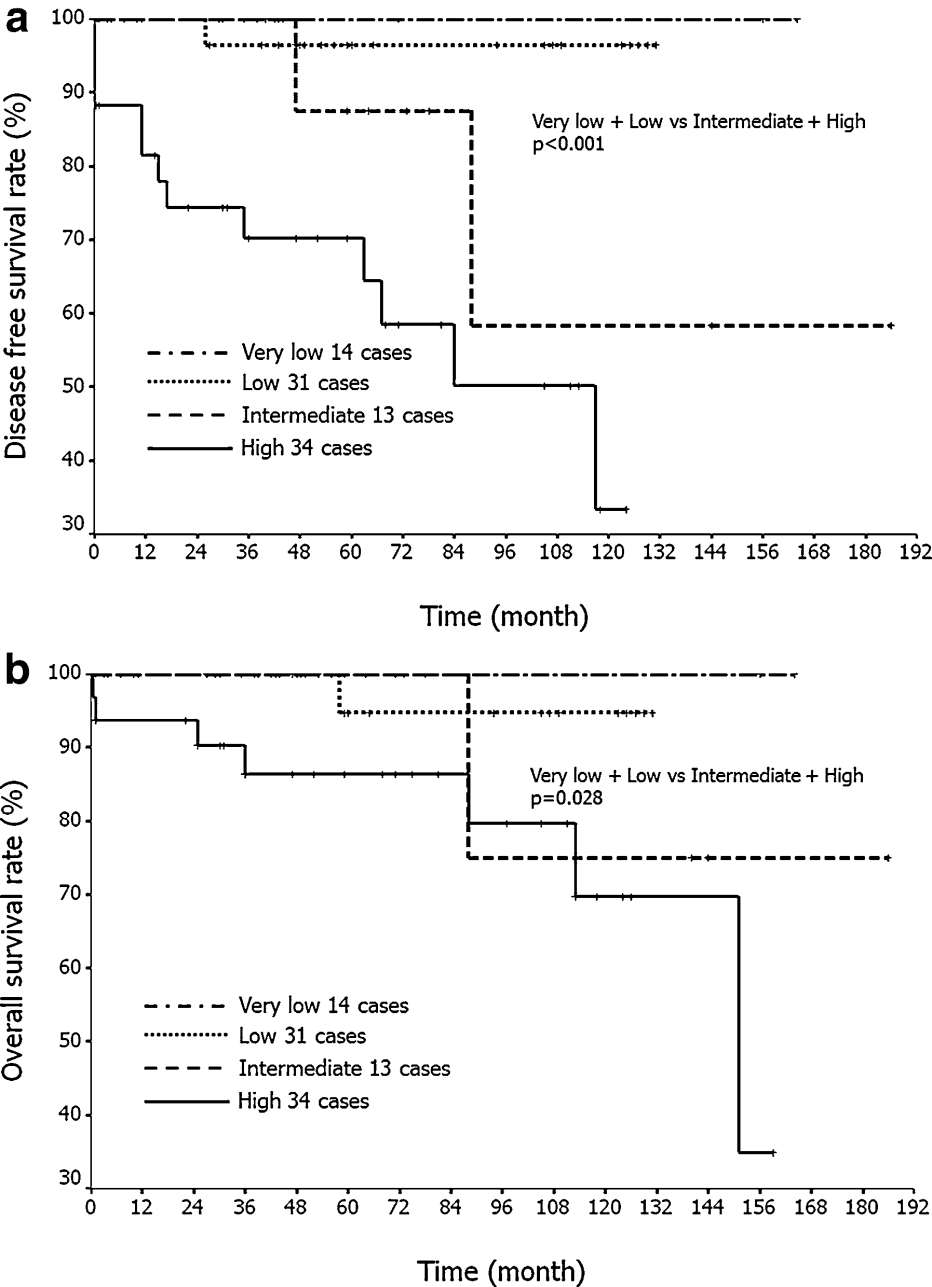
Kaplan–Meier analysis based on the risk classification (modified NIH consensus criteria). Intermediate-risk or high-risk tumors show significantly shorter disease free survival (**a**: P < 0.001) and overall survival (**b**: P = 0.028) compared to very low-risk or low-risk tumors. Disease-free survival rate: very low vs. low, P = 0.55; low vs. intermediate, P = 0.108; very low vs. intermediate, P = 0.225; intermediate vs. high, P = 0.121; very low vs. high, P = 0.015. Overall survival rate: very low vs. low, P = 0.646; low vs. intermediate, P = 0.515; very low vs. intermediate, P = 0.387; intermediate vs. high, P = 0.322; very low vs. high, P = 0.065

Clinicopathological information from all of the cases is summarized in Additional file 1: Table S1. The tumors occurred in 54 men and 38 women. The median tumor size was 4.2 cm (ranging from 2.9 to 7.0 cm). Fourteen, 31, 13, and 34 patients were classified as being at very low-, low-, intermediate-, and high-risk, respectively. Univariate analysis revealed that tumor size, presence of necrosis, and higher mitotic rate (>5/50 high-power fields [HPFs]) were associated with shorter disease free survival, and that tumor location (extra-gastric origin), tumor size and higher mitotic rate (>5/50 HPFs) were associated with shorter overall survival (Table 1). Furthermore, cases at intermediate- or high-risk showed significantly shorter DFS (P < 0.001) and OS (P = 0.028) compared to those at very low- or low-risk (Fig. 1a, b).

**Table 1:** Prognostic factors by univariate and multivariate analysis

Factor	DFS				OS		
	Number of	Univariate analysis	Multivariate analysis (Cox regression)		Univariate analysis	Multivariate analysis (Cox regression)	
	cases	log-rank test p value	Hazard ratio (95 % CI)	p value	log-rank test p value	Hazard ratio (95 % CI)	p value
Age							
≥60	51	0.278			0.152		
<60	41						
Age							
≥65	39	0.070			0.092		
< 65	53						
Sex							
F	38	0.436			0.571		
M	54						
Site							
Stomach	66	0.068			0.035	−^a^	−^a^
Others	26						
Size							
>5 cm	40	<0.001	14.02 (1.764−111.4)	0.013	0.005	4.488 (0.501−40.229)	0.180
≤5 cm	52						
Necrosis							
+	29	0.013	−^a^	−^a^	0.067		
–	63						
Mitosis							
>6	30	<0.001	5.644 (1.563−20.37)	0.008	0.001	7.531 (0.830−68.332)	0.073
≤5	62						
Risk Classification							
Very low or low	45	<0.001	−^a^	−^a^	0.028	−^a^	−^a^
Int or high	47						

^a^These factors were not selected in multivariate analysis

**Table 2:** Correlation between telomere dysregulation and clinicopathological features

Parameters	Telomere dysregulation		
	+	–	p value
Age			0.712
Median (range)	61.0 (49.5−65.5)	62.0 (51.0−68.0)	
Sex			0.372
F	10	28	
M	10	44	
Location			0.015
Stomach	10	56	
Others	10	16	
Size (cm)			0.966
Median (range)	4.0 (2.8−7.0)	4.4 (3.0−7.0)	
Mitosis/50HPF			0.514
≤5	13	49	
6−10	5	11	
>10	2	12	
Risk classification			0.854
Very low	4	10	
Low	6	25	
Intermediate	2	11	
High	8	26	
Necrosis			0.705
+	7	22	
–	13	50	
Metastasis or recurrence^a^			0.315
+	5	11	
–	14	60	
Total	20	72	

^a^Because two cases (cases 24 and 28) had residual tumor at the initial operation, these cases were excluded for analysis

Multivariate analysis revealed that tumor size and higher mitotic rate as independent prognostic factors for DFS. However, in this series of GISTs none of the clinicopathological parameters including risk classification was identified as independent prognostic factor for OS.

### *TERT* promoter hot spot mutations

**Fig. 2:**
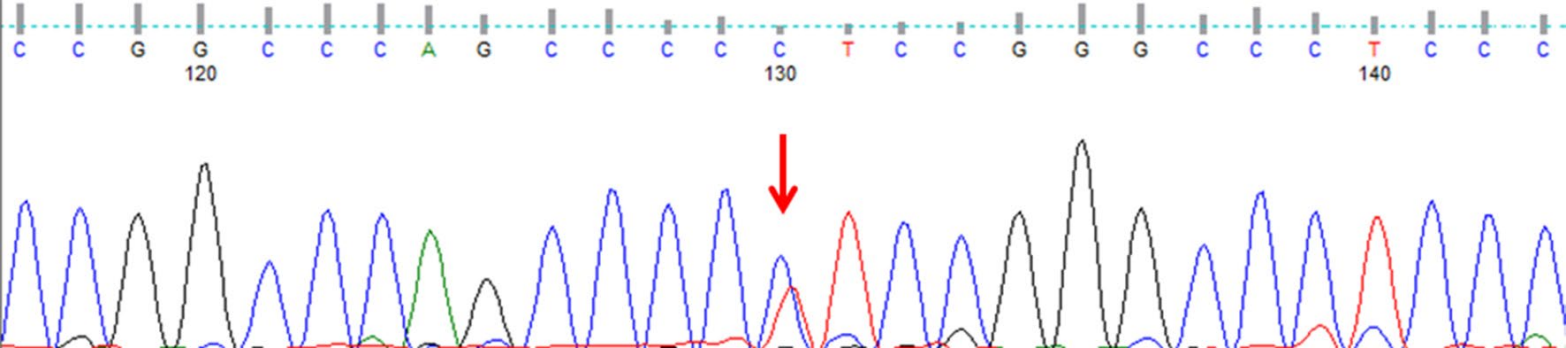
*TERT* promoter mutations in gastrointestinal stromal tumors (GISTs). *TERT* promoter hot spot mutation (C228T) detected in case #27

In this study, *TERT* promoter hot spot mutations were detected in only two cases (2 %), both of which were heterozygous C228T mutations (Fig. 2). One (case #17) of the two was classified as low-risk, and it was resected simultaneously with an esophageal carcinoma. The patient died of brain bleeding associated with multiple metastases of esophageal carcinoma 105 months after surgery. The other case (case #27) was classified as high-risk and it recurred locally 47 months after surgery, and the patient subsequently developed multiple liver metastases. The final outcome of this patient remained unknown, because the patient was transferred to another hospital 97 months after surgery.

### Immunohistochemistry for DAXX and ATRX

**Fig. 3:**
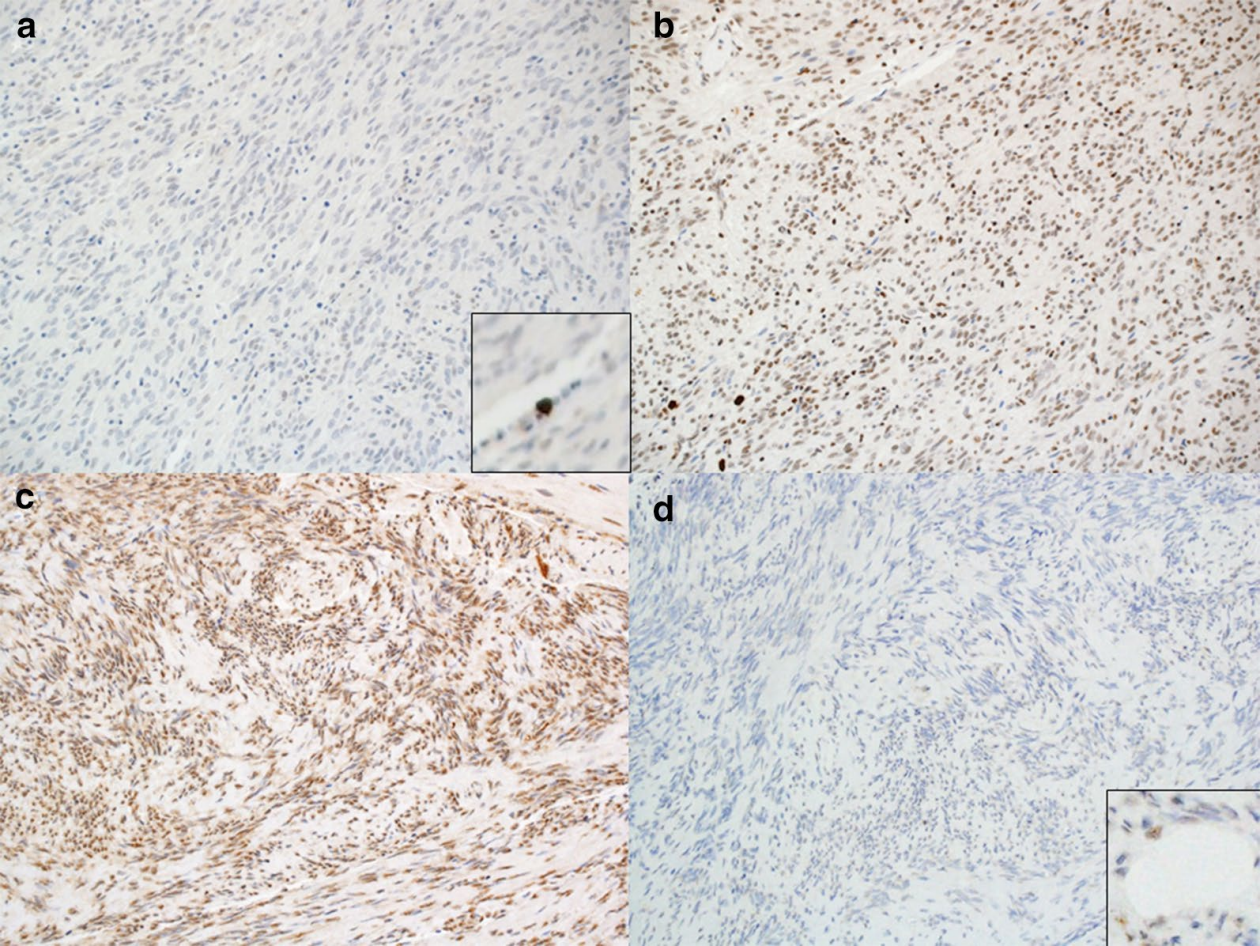
Immunohistochemistry for ATRX and DAXX. **a**, **b** In case #50, negative nuclear staining for ATRX (**a**) despite strong nuclear expression for DAXX **b**. **c**, **d** In case #86, only ATRX expression is observed (**c**). DAXX expression is lost (**d**). Positive staining of vascular endothelial cells are shown in the *insets* of **a** and **d**

Sixteen (17.4 %) and three cases (3.3 %) were negatively stained for ATRX and DAXX, respectively (Fig. 3a–d). All but one ATRX-negative case demonstrated nuclear staining for DAXX. Loss of expression of both ATRX and DAXX was observed in one case. Except for this case, *TERT* promoter hot spot mutations and loss of expression of either ATRX or DAXX occurred in a mutually exclusive fashion in our series of GISTs.

### Clinicopathological and survival analysis of telomere dysregulation

**Fig. 4:**
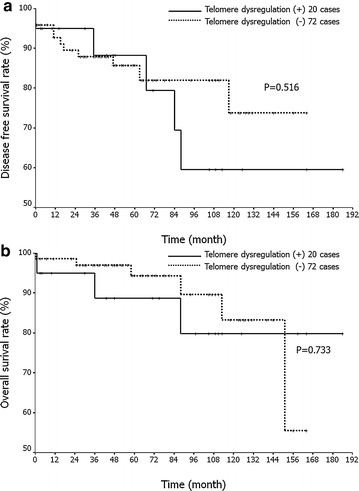
Prognostic impact of telomeres dysregulation modulated by loss of ATRX/DAXX expression or *TERT* promoter. **a**, **b** Kaplan–Meier analysis associated with disease-free (**a**) and overall (**b**) survival rates in gastrointestinal stromal tumors (GISTs). There are no significant differences in overall survival (P = 0.733) or disease-free survival (P = 0.516) according to the status of telomere dysregulation

Given that any cases with *TERT* promoter mutations or loss of either ATRX or DAXX expression were regarded as exhibiting telomere dysregulation, we examined the relationship between telomere dysregulation and clinicopathological features including age, sex, tumor size, tumor location, risk classification, presence of necrosis, and occurrence of metastasis or recurrence. Telomere dysregulation was frequently seen in GISTs of non-gastric origin (P = 0.015). However, the presence of telomere dysregulation did not statistically correlate with any other clinicopathological characteristics (Table 2). We also investigated associations of telomere dysregulation with DFS or OS. However, no significant differences were observed in OS (P = 0.733) or DFS (P = 0.516) according to the status of telomere dysregulation (Fig. 4a, b).

## Discussion

It is sometimes not easy to predict the clinical outcome of GIST patients, and intensive efforts have been made to find molecular biomarkers in addition to the classical clinicopathlogical risk factors (Joensuu et al. 2012; Miettinen and Lasota 2006). Few molecular biomarkers for predicting the malignant potential of tumors and patients’ prognosis in GISTs have been reported (Suehara et al. 2008; Kubota et al. 2013).

Recently, the impact of telomere dysregulation on aggressive behavior has been reported in many malignancies (Shay and Bacchetti 1997; Horn et al. 2013; Koelsche et al. 2013; Landa et al. 2013; Nault et al. 2013; Akaike et al. 2015; Saito et al. 2016). Two mechanisms are involved in telomere dysregulation: telomerase activation and ALT. Telomerase function can be activated by recurrent hot spot mutations (C228T and C250T) in the promoter region of *TERT* (Reddel 2014), resulting in an increase in *TERT* transcriptional activity. Therefore, we first examined the frequency of *TERT* promoter mutations in GISTs. To date, three research teams have reported information on *TERT* promoter mutations in GISTs (Campanella et al. 2015; Killela et al. 2013; Vinagre et al. 2013). Two of the three studies could not identify any *TERT* promoter mutations in their small cohorts (Killela et al. 2013; Vinagre et al. 2013). Campanella et al. (2015) examined the frequency of *TERT* promoter mutations in a large series of GISTs, and they found somatic mutations at a frequency of 3.8 % (5/130). In this study, we analyzed *TERT* promoter mutations (C228T and C250T) as well as expression of both DAXX and ATRX by immunohistochemistry in 92 cases of GISTs. Two of the 92 cases (2.2 %) had heterozygous C228T *TERT* promoter mutations. This frequency is very similar to that reported in a previous large study (Campanella et al. 2015).

ALT has been shown to be another mechanism for telomere lengthening that is independent of telomerase activity and is found in 10–15 % of cancers. Recently, it was discovered that PanNETs with the ALT phenotype had inactivation of either ATRX or DAXX (Heaphy et al. 2011; Jiao et al. 2011). Therefore, loss of the ATRX/DAXX dimer was suggested to be an important event in creating ALT-positive tumors (Heaphy et al. 2011). It has been recently demonstrated that loss of ATRX expression is highly correlated with the ALT phenotype in leiomyosarcomas (Liau et al. 2015). Moreover, previous studies reported that the ALT phenotype has prognostic implications in sarcomas, such as leiomyosarcomas, osteosarcomas, and liposarcomas (Liau et al. 2015; Ulaner et al. 2003; Venturini et al. 2010). However, the impact of the ALT phenotype on the clinicopathological features of GISTs has not been elucidated. In this study, 16 of 92 cases (17.4 %) showed loss of ATRX expression, and three of 92 cases (3.3 %) showed loss of DAXX expression. Loss of expression of these two proteins was mutually exclusive except in one case. Our two cases with *TERT* promoter mutations were detected in cases with preserved expression of DAXX and ATRX.

A previous study failed to find any correlation between *TERT* promoter mutations and clinicopathological factors in GISTs (Campanella et al. 2015); however, we found that the ALT phenotype was significantly associated with extra-gastric tumor origin. Furthermore, although telomere dysregulation was not associated with adverse outcomes in GISTs, it should be noted that telomere dysregulation was frequently observed in patients with GISTs of non-gastric origin, who have a worse overall survival rate compared to those with gastric GISTs. GISTs of non-gastric origin have commonly a poorer prognosis than those of gastric origin (Joensuu et al. 2012; Miettinen and Lasota 2006). Therefore, these findings suggest that a poorer prognosis of non-gastric GISTs might be affected by telomere dysfunction. Although we found that telomere dysregulation did not have a prognostic impact in GISTs of non-gastric origin (data not shown), some previous studies demonstrated the associations between telomerase activity and malignant or metastatic potential in GISTs by TRAP analysis (Sakurai et al. 1998; Kawai et al. 2005; Wang and Kou 2007). Regarding to our two *TERT* promoter mutated cases, one case was classified as low-risk and the other case as high-risk. Unfortunately, we cannot examine telomerase activity for those two cases because of lack of frozen section. To affirm association between telomerase activity and *TERT* promoter mutations in GIST, larger studies with frozen materials are required.

In conclusion, *TERT* promoter mutations were rare in Japanese GISTs, similar to what has been previously shown in a European study (Campanella et al. 2015). Telomere dysregulation defined as either *TERT* promoter mutations or loss of ATRX/DAXX expression was frequently observed in GISTs of extra-gastric origin, but this phenotype was not associated with patient prognosis. These findings suggest that telomere maintenance by telomerase activation and ALT might not play an important role in tumor progression of GISTs. However, much larger number of cases by multicenter studies are required to conclude the frequency of telomere dysregulation and its prognostic impacts in GISTs.

## Additional file

10.1186/s40064-015-1606-2 Clinicopathologic information of the 92 GIST cases.
